# Knowledge, confidence, and practices of clinical associates in the management of mental illness

**DOI:** 10.4102/sajpsychiatry.v29i0.2074

**Published:** 2023-10-26

**Authors:** Saiendhra V. Moodley, Jacqueline Wolvaardt, Christoffel Grobler

**Affiliations:** 1 School of Health Systems and Public Health, Faculty of Health Sciences, University of Pretoria, Pretoria, South Africa

**Keywords:** clinical associates, knowledge, confidence, practices, South Africa, mental health mental illness

## Abstract

**Background:**

Additional human resources are needed to provide mental health services in underserved areas in South Africa (SA). Clinical associates, the mid-level medical worker cadre in SA, could potentially be used to deliver these services.

**Aim:**

The study explored the self-reported knowledge, confidence, and current practices of clinical associates related to mental health assessment and management.

**Setting:**

South Africa.

**Methods:**

A cross-sectional study was conducted. The link to the electronic questionnaire was distributed to clinical associates via databases and social media. Data were analysed with Stata v17.

**Results:**

Of the 209 participants, 205 (98.1%) indicated they had training on management of patients with mental illness during their undergraduate degree and 192 (91.9%) had a mental health rotation. Few (10.7%) had any additional mental health training. Most participants rated their knowledge of priority mental disorders as ‘good’ or ‘excellent’. Only 43.2% of the participants felt quite or very confident to perform a mental health examination. Participants who felt quite or very confident to manage patients presenting with suicide risk, aggression, and confusion were 44.9%, 46.9% and 53.1%, respectively. Factors associated with a confidence score of 75% and higher were male gendered, working in Gauteng or Northern Cape provinces, and in a rural area. The majority of participants were already involved in mental health assessment and management in their current work.

**Conclusion:**

Clinical associates have a contribution to make in mental health service provision, but this may need to be supplemented by additional practical training.

**Contribution:**

Potential gaps in training have been identified.

## Background

Globally, the supply of health workers is outstripped by demand.^1^ The global health workforce crisis resulting from supply deficits is exacerbated by distribution deficiencies between, and within, countries.^2,3^ Task shifting and task sharing approaches involving mid-level medical workers have been one of the strategies used to address the shortage of medical doctors. These health workers undergo a shorter period of training than medical doctors but carry out some of the diagnostic and treatment functions usually performed by doctors.^4,5,6,7^ The South Africa government took a decision to develop a mid-level medical worker cadre – the clinical associate – to address shortages of skilled health professionals at the district level and specifically in rural areas.^8^ Training of this cadre is done at three universities that graduate approximately 70 to 140 clinical associates annually.^8,9^ Students are placed in district hospitals early in the programme – with an integration of case-based learning and clinical involvement.^8^ There are currently 1797 clinical associates registered with the Health Professionals Council of South Africa (HPCSA).^10^ There is promising early evidence of them potentially addressing health workforce shortages in the public sector and rural areas.^9,11^

Clinical associates could potentially be utilised to deliver mental health services in underserved areas. The inequitable distribution of the specialist mental health workforce in South Africa is well documented.^12,13,14,15^ Approximately 20% of psychiatrists work full time in the public sector which serves the majority of the population.^15^ Five provinces in South Africa (Eastern Cape, Limpopo, Mpumalanga, North West, and Northern Cape) have less than 1.0 psychiatrist per 100 000 population compared to Gauteng and the Western Cape which has 2.6 psychiatrists and 5.0 psychiatrists per 100 000 population. respectively.^15^ Access to specialist mental health nurses in rural primary health care (PHC) facilities is also a challenge with one study reporting a ratio of 0.68 mental health nurses per 100 000 population.^13^ Given that non-specialist medical doctors and nurses are also in short supply in many of these areas, the use of clinical associates in mental health task sharing needs to be considered.

The scope of practice of clinical associates includes taking mental health history, performing a mental health examination, and mental health counselling.^16^ Participants in a recent study conducted among university- and facility-based staff involved in clinical associate training as well as clinical associate student representatives expressed support for a role for this cadre in providing mental health services.^17^ Potential roles for them were the immediate care of mental health patients in emergency settings, provision of basic inpatient care, follow up of patients in outpatient settings, and home visits.^17^ Similar cadres have been used to varying degrees to deliver mental services in several countries including Kenya, Malawi, the United Kingdom, and the United States of America (USA).^18,19,20,21,22,23^

A study involving all three universities offering the degree confirmed that mental health forms part of the training in all three programmes.^24^ The training ranges from two to four weeks in the final year of study with a mix of theoretical and practical components varying between the programmes.^24^ The authors concluded that there may be limitations in the roles clinical associates could play in mental health based on the training gaps identified.^24^ What is not known is the readiness of clinical associates to take on mental health task sharing and their level of current involvement in providing mental health services. In this study, we explored clinical associates’ knowledge, confidence, and current practices related to mental health assessment and management. In addition, we determined the demographic, training, and employment factors associated with participants’ confidence to perform a mental health assessment and manage patients with mental illness.

## Research methods and design

### Study design

A cross-sectional study utilising an electronic questionnaire was conducted between 01 December 2021 and 31 July 2022.

### Study population and sampling

The study population were clinical associates based in South Africa. In order to be included in the study, they needed to have been qualified for a minimum of 6 months at the time of the survey. Clinical associates who had emigrated from South Africa, had subsequently qualified with a medical degree or who were pursuing a medical degree at the time of the survey were excluded. The survey did not involve sampling as all clinical associates who could be reached were requested to participate.

### Measurement tools

An electronic questionnaire was developed using Qualtrics software. The questionnaire included sections on self-assessed mental health knowledge, confidence to carry out various aspects of a mental health assessment, and their current involvement in mental health work. The knowledge, confidence, practice, and interest items that used scales underwent expert validation for representativeness, clarity, and relevance.^25^ This process was followed by individual cognitive interviews with five clinical associates to ensure respondents interpret items as intended by the researcher.^25^ Detail on the expert validation and cognitive interview processes followed has been detailed by Moodley et al^26^ previously.

### Data collection

The Professional Association of Clinical Associates in South Africa (PACASA) assisted with distributing the survey information and link to the Qualtrics questionnaire using their available platforms including social media. In addition, two of the three universities distributed the survey information and the questionnaire link to their alumni. Potential participants who clicked on the link had to complete five questions related to the inclusion criteria. If they met the criteria, they were then provided with a participant information leaflet and needed to provide electronic informed consent prior to the first question. In order to improve the response rate, incentives were offered in the form of five gift vouchers to the value of R1000.00 each awarded to five randomly-selected respondents. The available functionality in Qualtrics was utilised to prevent individuals from completing the survey more than once. As the survey was anonymous, an end-of-survey redirect was used for completion of contact information for the incentives ensuring that the contact information was not linked to the questionnaire.^27^

### Data management and analysis

The data were downloaded from Qualtrics and stored on a password-protected computer with a backup stored in a password-secured Cloud account. The data were imported into Stata version 17 (Statacorp; http://www.stata.com) for analysis. Proportions were calculated for the demographic variables and for each of the knowledge, confidence, and practice items that used scales. Each knowledge item was scored from 0 to 4, as was each confidence item. A knowledge score for each participant was calculated by adding the scores of the 12 knowledge items and a confidence score was calculated by adding the scores of the 27 confidence items. Categories were created for the knowledge and confidence scores based on the scoring equivalent of 75% or more, 50% – 74%, 25% – 49% and 0% – 24%. Scoring 81 or more out of a possible 108 was, therefore, regarded as a high confidence score. Participants were categorised as either having a high confidence score or not, which was our main outcome measure. A bivariate analysis of high confidence score and sociodemographic, employment, and training variables was conducted. Variables with *p* < 0.25 were included in our initial multivariate logistic regression model. A manual backwards stepwise elimination process was used to arrive at a final model.

### Ethical considerations

The study had ethical approval from the University of Pretoria Faculty of Health Sciences Research Ethics Committee (778/2020). Informed consent was obtained from all participants.

## Results

A total of 216 individuals who met the inclusion criteria consented to participate in the study. Of these, two individuals provided no responses and five individuals provided only some demographic information and no other responses. These seven individuals were dropped from the analysis. A total of 209 participants were, therefore, included in the analysis. Using the HPCSA register of clinical associates as a proxy, this represents approximately 11.6% (209/1797) of clinical associates in the country.^10^

### Demographic and employment characteristics

Just under half of participants (49.8%) were aged between 25 and 29 years (Table 1). The majority of participants (72.1%) were female. Almost half of participants (49.5%) indicated that they currently worked in Gauteng province. Most participants (57.7%) indicated that they worked in an urban area. Roughly one-third of participants (34.6%) were employed by a provincial department of health, while 28.8% were employed by a private health facility or private medical practice. The most common work settings of the participants were district hospitals (26.0%), private general practice (17.8%), and academic institutions (13.0%).

**TABLE 1:** Demographic and employment characteristics of participants.

Characteristic (*N*)	Categories	*n*	%
Age (*N* = 209)	20–24 years	46	22.0
	25–29 years	104	49.8
	30–34 years	45	21.5
	35–39 years	13	6.2
	40 years and older	1	0.5
Gender (*N* = 208)	Female	150	72.1
	Male	56	26.9
	Prefer not to say	2	1.0
Province of current work (*N* = 208)	Eastern Cape	22	10.6
	Free State	9	4.3
	Gauteng	103	49.5
	KwaZulu Natal	16	7.7
	Limpopo	19	9.1
	Mpumalanga	17	8.2
	Northern Cape	7	3.4
	North West	14	6.7
	Western Cape	1	0.5
Municipality of current work (*N* = 193)	District	102	52.8
	Metropolitan	91	47.2
Area of current work (*N* = 208)	Rural	88	42.3
	Urban	120	57.7
Current employer (*N* = 208)	Provincial Department of Health	72	34.6
	Private health facility and/or private medical practice	60	28.8
	Non-governmental organisation	22	10.6
	Academic institution	40	19.2
	Self-employed	1	0.5
	Unemployed	8	3.8
	Other	5	2.4
Work setting (*N* = 208)	Primary health care clinic	22	10.6
	Community health centre	14	6.7
	District hospital	54	26.0
	Regional hospital	3	1.4
	Tertiary or central hospital	6	2.9
	Private general practice	37	17.8
	Private specialist practice	6	2.9
	Private hospital	14	6.7
	Academic institution	27	13.0
	Unemployed	9	4.3
	Other	16	7.7

### Training characteristics

Fewer participants had completed their Bachelor of Medicine in Clinical Practice (BMCP) degree at Walter Sisulu University (WSU) (South Africa) (13.9%) than Bachelor of Clinical Medical Practice (BCMP) degrees at University of Pretoria (43.1%) or University of the Witwatersrand (43.1%) (Table 2). Only two participants (1.0%) indicated they had not received training on assessing patients with mental illness during their BCMP or BMCP degree and four participants (1.9%) indicated they had not received training on managing patients with mental illness. Most participants (91.9%) confirmed they had a mental health rotation during their degree. Just over one-tenth of the participants (10.7%, 22/206) indicated they had received additional mental health-related training after completing their degree.

**TABLE 2:** Characteristics of participants’ undergraduate clinical associate training.

Characteristic (*N*)	Categories	*n*	%
University (*N* = 209)	University of Pretoria	90	43.1
	University of Witwatersrand	90	43.1
	Walter Sisulu University	29	13.9
	Other	0	0.0
Length of time since qualifying (*N* = 209)	Less than 3 years	46	22.0
	Between 3 and 6 years	96	45.9
	More than 6 years	67	32.1
Received training on assessment of patients with mental illness (*N* = 208)	Yes	206	99.0
	No	2	1.0
Received training on management of patients with mental illness (*N* = 209)	Yes	205	98.1
	No	4	1.9
Mental health rotation formed part of the degree (*N* = 209)	Yes	192	91.9
	No	17	8.1
Length of mental health rotation (*N* = 204)	No rotation	17	8.3
	1–2 weeks	29	14.2
	3–4 weeks	100	49.0
	5–6 weeks	38	18.6
	7–8 weeks	10	4.9
	More than 8 weeks	10	4.9
Site of mental health rotation (*N* = 205)	No rotation	17	8.3
	Primary health care clinic only	2	1.0
	Community health centre only	0	0.0
	District hospital only	79	38.5
	Regional hospital only	55	26.8
	Tertiary or central hospital only	28	13.7
	Specialised psychiatric hospital only	5	2.4
	Other	0	0.0
	Combination of two or more of the above sites	19	9.3

### Mental health knowledge

The participants’ self-assessment of their knowledge of various mental disorders that are considered important in the South African context are shown in Table 3. Approximately 70% of the participants rated their knowledge of substance use disorders (69.7%) and depressive disorders (70.2%) as good or excellent, while only 50.5% rated their knowledge of dementia as good or excellent, and even fewer rated their knowledge of attention-deficit hyperactivity disorder (ADHD) (29.5%) as good or excellent. With respect to their knowledge of the management of common mental health presentations, most participants rated their knowledge of the confused patient (66.3%), suicide risk (59.0%), and the aggressive patient (55.6%) as good or excellent.

**TABLE 3:** Participants’ self-assessment of their knowledge of mental health conditions and presentations.

Item	Very poor		Poor		Fair		Good		Excellent	
	*n*	%	*n*	%	*n*	%	*n*	%	*n*	%
**Knowledge of mental health conditions**										
Depressive disorders (*N* = 198)	3	1.5	5	2.5	51	25.8	95	48.0	44	22.2
Substance use disorders (*N* = 201)	0	0.0	6	3.0	55	27.4	100	49.8	40	19.9
Anxiety disorders (*N* = 200)	1	0.5	12	6.0	58	29.0	97	48.5	32	16.0
Post-traumatic stress disorder (*N* = 201)	6	3.0	11	5.5	66	32.8	87	43.3	31	15.4
Schizophrenia (*N* = 198)	4	2.0	14	7.1	78	39.4	72	36.4	30	15.2
Dementia (*N* = 202)	5	2.5	21	10.4	74	36.6	78	38.6	24	11.9
Acute-stress disorder (*N* = 199)	5	2.5	19	9.5	69	34.7	86	43.2	20	10.1
Bipolar disorders (*N* = 202)	2	1.0	13	6.4	80	39.6	90	44.6	17	8.4
Attention-deficit hyperactivity disorder (*N* = 200)	10	5.0	36	18.0	95	47.5	51	25.5	8	4.0
**Knowledge of management of mental health presentations**										
Aggressive patient (*N* = 196)	4	2.0	13	6.6	70	35.7	77	39.3	32	16.3
Confused patient (*N* = 199)	4	2.0	11	5.5	52	26.1	103	51.8	29	14.6
Suicide risk (*N* = 200)	2	1.0	14	7.0	66	33.0	90	45.0	28	14.0

Of the 188 participants who answered all 12 knowledge questions, the knowledge score ranged from 9 to 48. The median score was 31, and the mean was 31.26 (s.d. = 7.47). Three participants (1.6%) scored less than 12. Twenty participants (10.6%) scored between 12 and 23. The majority of participants (*n* = 113, 60.1%) scored between 24 and 35. Over a quarter of participants (*n* = 52, 27.7%) scored 36 and higher.

### Confidence in mental health assessment and management

With respect to conducting a mental health assessment on individuals presenting with mental health symptoms, there was some variation in confidence in conducting the different components of the assessment (Table 4). Approximately half of participants (50.3%) felt quite or very confident taking a mental health history, but only 43.2% of the participants felt quite or very confident in carrying out a mental health examination. A greater proportion of participants were quite or very confident doing a physical examination (59.1%) and ordering relevant investigations (61.2%).

**TABLE 4:** Participants’ confidence in mental health assessment and management.

Item	Not at all confident		Slightly confident		Moderately confident		Quite confident		Very confident	
	*n*	%	*n*	%	*n*	%	*n*	%	*n*	%
**Assessment of an individual presenting with mental health symptoms**										
Taking a mental health history (*N* = 199)	4	2.0	19	9.5	76	38.2	73	36.7	27	13.6
Doing a mental health examination (*N* = 199)	6	3.0	30	15.1	77	38.7	56	28.1	30	15.1
Assessing cognitive functioning using a suitable cognitive screening test (*N* = 198)	7	3.5	30	15.2	60	30.3	54	27.3	47	23.7
Doing a physical examination (*N* = 198)	5	2.5	18	9.1	58	29.3	63	31.8	54	27.3
Ordering relevant investigations (*N* = 196)	7	3.6	17	8.7	52	26.5	65	33.2	55	28.1
**Management of mental health presentations**										
Confused patient (*N* = 194)	9	4.6	25	12.9	57	29.4	75	38.7	28	14.4
Aggressive patient (*N* = 194)	10	5.2	29	14.9	64	33.0	64	33.0	27	13.9
Suicide risk (*N* = 196)	8	4.1	30	15.3	70	35.7	63	32.1	25	12.8
Patient suspected to be exposed to traumatic event(s) (*N* = 194)	13	6.7	26	13.4	71	36.6	60	30.9	24	12.4
**Prescribing pharmacological treatment for:**										
A depressive disorder (*N* = 193)	12	6.2	33	17.1	59	30.6	68	35.2	21	10.9
An anxiety disorder (*N* = 193)	12	6.2	35	18.1	64	33.2	56	29.0	26	13.5
A substance use disorder (*N* = 193)	22	11.4	32	16.6	72	37.3	36	18.7	31	16.1
Schizophrenia (*N* = 191)	29	15.2	30	15.7	65	34.0	44	23.0	23	12.0
**Providing counselling to a patient with:**										
A depressive disorder (*N* = 194)	6	3.1	27	13.9	61	31.4	62	32.0	38	19.6
An anxiety disorder (*N* = 193)	10	5.2	26	13.5	64	33.2	61	31.6	32	16.6
A substance use disorder (*N* = 194)	7	3.6	25	12.9	66	34.0	57	29.4	39	20.1
Schizophrenia (*N* = 193)	29	15.0	39	20.2	70	36.3	34	17.6	21	10.9
Suicide risk (*N* = 191)	10	5.2	26	13.6	62	32.5	62	32.5	31	16.2
**Provide counselling to the family of a patient with:**										
A depressive disorder (*N* = 194)	7	3.6	29	14.9	62	32.0	63	32.5	33	17.0
An anxiety disorder (*N* = 192)	12	6.3	23	12.0	66	34.4	64	33.3	27	14.1
A substance use disorder (*N* = 192)	5	2.6	29	15.1	49	25.5	73	38.0	36	18.8
Schizophrenia (*N* = 191)	15	7.9	38	19.9	62	32.5	53	27.7	23	12.0
Suicide risk (*N* = 190)	9	4.7	26	13.7	58	30.5	67	35.3	30	15.8
**Other**										
Sedating a patient who is aggressive or violent (*N* = 194)	15	7.7	25	12.9	62	32.0	47	24.2	45	23.2
Completion of the relevant forms for 72-h observation of a mental health patient (*N* = 190)	29	15.3	34	17.9	48	25.3	42	22.1	37	19.5
Managing common side effects from psychiatric medication (*N* = 191)	22	11.5	34	17.8	67	35.1	51	26.7	17	8.9
Managing serious adverse events from emergency psychiatric medication (*N* = 191)	37	19.4	39	20.4	62	32.5	41	21.5	12	6.3

More participants felt quite or very confident managing patients presenting with confusion (53.1%) than patients presenting with suicide risk (44.9%) or aggression (46.9%). The participants were also asked to rate their confidence in carrying out mental health management tasks for specific conditions. Less than half of participants were quite or very confident to prescribe pharmacological treatment for all of the conditions listed. More than half of participants (51.5%) were quite or very confident to provide counselling to a patient with depressive disorder.

While 47.4% of the participants were quite or very confident sedating an aggressive or violent patient, only 27.7% of the participants were quite or very confident managing serious adverse events from emergency psychiatric medication. Only 41.6% of the participants were quite or very confident completing the relevant forms for 72 h observation of a mental health patient.

**FIGURE 1:**
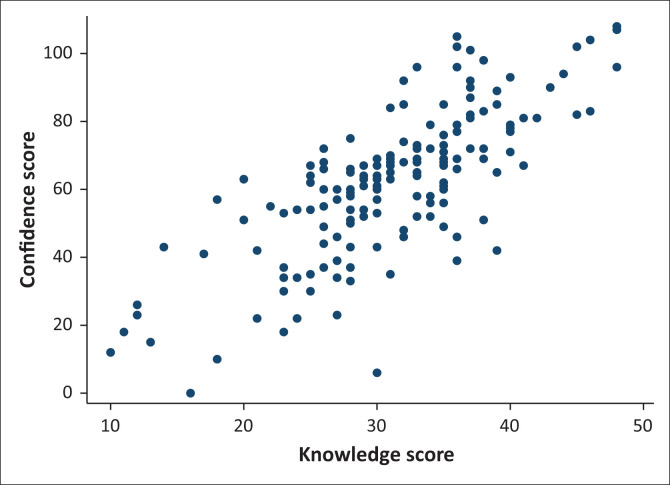
Correlation between confidence score and knowledge score (*N* = 161).

A total of 169 participants answered all 27 confidence questions. The median score was 64 (range 0–108). The mean score was 62.18 with a s.d. of 21.89. Just over one-fifth of participants (*n* = 34, 20.1%) scored 81 and higher with just under half of participants (*n* = 84, 49.7%) scoring between 54 and 80. Fewer participant (*n* = 39, 23.1%) scored 27 to 53 with only 12 participants (7.1%) scoring between 0 and 26. There was high correlation between confidence score and knowledge score (Figure 1) with a correlation co-efficient of 0.76.

With respect to demographic, employment, and training characteristics associated with a high confidence score (defined as a minimum of 81/108 i.e., 75% and higher), bivariate analysis identified a number of variables with a *p* < 0.25 to include in the multivariate model. These were age, gender, province of current work, area of current work, current employer, work setting, university, length of mental health rotation, and site of mental health rotation. Following logistic regression, the variables found to be significantly associated with a high confidence score were gender, province of current work, and area of current work (Table 5). Being male, working in Gauteng province and Northern Cape province, and working in a rural area significantly increased the odds of a high confidence score.

**TABLE 5:** Multivariate model of characteristics associated with a high confidence score.

Characteristic	Odds ratio	95% Confidence interval	*p*-value
**Gender**			
Male	3.45	1.49–7.97	0.004
**Province of current work**			
Gauteng	3.99	1.02–15.68	0.047
Northern Cape	17.82	2.06–154.23	0.009
**Area of current work**			
Rural	4.64	1.25–17.29	0.022

### Current practices

The current work of most participants (Table 6) included taking a mental health history and doing a mental health examination with 35.6% of the participants indicating that their current work ‘often’ involved taking a mental health history from patients suspected of a substance use disorder and 34.1% indicating that their current work ‘often’ involved doing a mental health examination on patients suspected of a substance use disorder. The majority of participants provide counselling to patients with mental illness as part of their current work with 31.5% indicating they ‘often’ provide counselling to patients with depression. More than one-fifth of the participants indicated they ‘often’ prescribed medication for depressive disorders (27.8%), anxiety disorders (24.6%), substance use disorders (25.7%) and suicide risk (21.3%). Current work included ‘often’ sedating aggressive or violent patients for 19.9% of participants. Current work did not involve completing the relevant forms for 72 h observation of a mental health patient for 61.6% of the participants.

**TABLE 6:** Mental health asssessment and management as part of current work of participants.

Item	Never		Sometimes (four times or less per month)		Often (five times or more per month)	
	*n*	%	*n*	%	*n*	%
**Taking a mental history from patients suspected of having:**						
A depressive disorder (*N* = 174)	42	24.1	81	46.6	51	29.3
An anxiety disorder (*N* = 173)	48	27.7	83	48.0	42	24.3
A substance use disorder (*N* = 174)	44	25.3	68	39.1	62	35.6
Schizophrenia (*N* = 173)	71	41.0	66	38.2	36	20.8
Suicidal risk (*N* = 171)	51	29.8	74	43.3	46	26.9
**Doing a mental health examination on patients suspected of having:**						
A depressive disorder (*N* = 173)	45	26.0	79	45.7	49	28.3
An anxiety disorder (*N* = 173)	48	27.7	83	48.0	42	24.3
A substance use disorder (*N* = 173)	47	27.2	67	38.7	59	34.1
Schizophrenia (*N* = 174)	77	44.3	61	35.1	36	20.7
Suicidal risk (*N* = 171)	54	31.6	67	39.2	50	29.2
**Doing a physical examination on patients suspected of having:**						
A depressive disorder (*N* = 173)	50	28.9	71	41.0	52	30.1
An anxiety disorder (*N* = 172)	47	27.3	81	47.1	44	25.6
A substance use disorder (*N* = 173)	49	28.3	64	37.0	60	34.7
Schizophrenia (*N* = 174)	70	40.2	66	37.9	38	21.8
Suicidal risk (*N* = 169)	49	29.0	68	40.2	52	30.8
**Ordering special investigations in patients suspected of having:**						
A depressive disorder (*N* = 173)	68	39.3	62	35.8	43	24.9
An anxiety disorder (*N* = 170)	72	42.4	64	37.6	34	20.0
A substance use disorder (*N* = 170)	62	36.5	60	35.3	48	28.2
Schizophrenia (*N* = 173)	83	48.0	53	30.6	37	21.4
Suicidal risk (*N* = 169)	71	42.0	59	34.9	39	23.1
**Providing counselling to patients with:**						
A depressive disorder (*N* = 168)	44	26.2	71	42.3	53	31.5
An anxiety disorder (*N* = 169)	42	24.9	83	49.1	44	26.0
A substance use disorder (*N* = 166)	42	25.3	72	43.4	52	31.3
Schizophrenia (*N* = 168)	68	40.5	63	37.5	37	22.0
Suicidal risk (*N* = 167)	50	29.9	67	40.1	50	29.9
**Providing counselling to the families of patients with:**						
A depressive disorder (*N* = 168)	59	35.1	75	44.6	34	20.2
An anxiety disorder (*N* = 168)	62	36.9	78	46.4	28	16.7
A substance use disorder (*N* = 168)	56	33.3	73	43.5	39	23.2
Schizophrenia (*N* = 167)	78	46.7	58	34.7	31	18.6
Suicidal risk (*N* = 165)	61	37.0	67	40.6	37	22.4
**Prescribing pharmacological treatment to patients with:**						
A depressive disorder (*N* = 169)	68	40.2	54	32.0	47	27.8
An anxiety disorder (*N* = 167)	60	35.9	66	39.5	41	24.6
A substance use disorder (*N* = 167)	76	45.5	48	28.7	43	25.7
Schizophrenia (*N* = 166)	88	53.0	47	28.3	31	18.7
Suicidal risk (*N* = 164)	76	46.3	53	32.3	35	21.3
**Other**						
Assessing the cognitive functioning of a patient with confusion using a suitable cognitive screening test (*N* = 165)	51	30.9	66	40.0	48	29.1
Sedating an aggressive or violent patient (*N* = 166)	77	46.4	56	33.7	33	19.9
Completing the relevant forms for 72-h observation of a mental health patient (*N* = 164)	101	61.6	36	22.0	27	16.5

## Discussion

In this study, we explored the readiness of clinical associates to take on mental health task sharing by determining clinical associates’ knowledge, confidence, and current practices related to mental health assessment and management. The study confirmed previous findings that mental health forms part of the curriculum of all three undergraduate programmes^24^ with virtually all of the participants in our study indicating they had received some training in mental health assessment and management as part of their undergraduate training. Seventeen participants indicated that they did not have a mental health rotation even though all three universities include this in their curricula.^24^ It is possible that these students were based at facilities where there was no mental health training even though the rotation existed on paper.

Most clinical associates indicated ‘good’ or ‘excellent’ knowledge of all the listed mental illnesses with the exception of ADHD. Deficiencies in ADHD knowledge in this study confirms previous findings of gaps with respect to training in childhood disorders that had been highlighted at two of the universities.^24^ The high levels of knowledge with respect to substance use disorder may in part be related to one of the universities having a focus on this and including an option to spend a week of their mental health rotation at a community-based substance use programme.^24^ The findings with regard to the high levels of knowledge of depression contrast with studies among non-specialist health professionals in Africa that report limited knowledge of depression.^28,29^ A study amongst PHC workers in South Africa and Zambia found moderate mental health literacy,^30^ while a study amongst non-specialist medical practitioners in the public and private sector in South Africa found adequate knowledge of mental illness.^31^ The use of a self-rating tool in the study may have meant that participants could have overestimated their knowledge.

While there was a strong correlation between the confidence score and knowledge scores, self-rated knowledge scores did not necessarily translate into confidence with mental health assessment and management. Almost 88% of the participants had a self-rated knowledge score of 50% or more, while approximately 70% of the participants had a confidence score of 50% or more. This finding concurs with a previous finding that most of the undergraduate practical training in mental health at two of the universities is by chance, thereby limiting their opportunity to apply their knowledge.^24^ Unsurprisingly, participants were more confident doing a physical examination and ordering relevant investigations which they would usually do in the normal course of their work rather than the mental health specific aspects of the assessment. They also appeared to be more confident managing confused patients which is more likely to be a medical diagnosis than aggression or suicide risk. Suicide risk assessment has previously been noted as a possible gap in their training.^24^

The number of participants with high levels of confidence in prescribing pharmacological treatment was relatively low. This could be potentially related to scope of practice as clinical associates are only permitted to prescribe medicines (under supervision of a medical practitioner) up to Schedule 4.^16^ Psychoactive medicines such as anti-depressants and sedatives are Schedule 5.^32^ Although all three universities include pharmacological management in their mental health curriculum, it is not clear how much of attention is given to the topic as there is only evidence of one university having a dedicated lecture.^24^ Managing side effects and adverse events from psychotropic medication also appeared to be a gap for many participants. With respect to depressive, anxiety and substance use disorders, it appeared that a greater number of participants had higher levels of confidence to provide counselling rather than prescribing pharmacological treatment. Given that clinical associates are likely to encounter aggressive or violent patients in emergency settings, it is worrying that less than half of participants had high levels of confidence to sedate such patients.

The authors found that a high confidence score was associated with gender. Males had significantly higher odds of a score of 75% (81/108) and higher in our multivariate model. A systematic review that explored the confidence gap between male and female medical students, residents, and faculty found that male self-reported scores in a number of areas including knowledge, skills, and procedural confidence were higher than females in 24 of 31 studies.^33^ In the remaining studies, there were no gender differences in five studies with only two studies finding higher scores among females than males.^33^ Vajapey et al.^33^ concluded that female health professionals perceive deficiencies with respect to their abilities more frequently than male counterparts, even though there is no difference in clinical performance. Gavinski et al.^34^ note that self-reported confidence does not necessarily mean a higher level of competence. Our findings suggest that the gender differences in confidence seen in medical and other health professionals may also be applicable to clinical associates.

High confidence scores were also associated with province of work. It is not clear why working in Gauteng province or the Northern Cape province would increase the odds of a high confidence score particularly as these provinces are very different. Gauteng province is South Africa’s economic hub with a high population density and the Northern Cape province is South Africa’s largest province by surface area with a low population density.^35^ The authors also found that working in a rural area significantly increased the odds of a high confidence score. This finding appears contradictory as Gauteng province, which was also associated with high confidence scores, is predominately an urban province. It should be noted that the finding with respect to Gauteng province was borderline significant with a *p*-value of 0.047 and a lower limit of the 95% confidence interval of 1.02. Lower confidence scores in urban areas other than Gauteng province could have resulted in the apparently contradictory findings. A potential explanation for higher confidence scores among clinical associates working in rural areas could be a greater opportunity for them to be involved in mental health assessment and management because of health workforce shortages in these areas.^36^

Findings indicate that a majority of participants are already doing work related to mental health assessment and management. This finding is not surprising taking into account the work settings of the participants and the high prevalence of mental illness in South Africa.^37,38^ A study amongst a similar cadre viz., physician assistants in the USA found they see and evaluate patients with mental illness on a regular basis with 62% of physician assistants doing so at least weekly.^23^ A large number of participants in our study indicated they were prescribing pharmacological treatment for mental illness. This reported practice is despite their scope of practice limiting them to prescribing no higher than Schedule 4 medication.^16^

The overall findings suggest that clinical associates generally have good self-assessed knowledge of mental health conditions and have opportunities to assess and manage mental health patients; but there appears to be a confidence ‘deficit’ in performing some key mental health tasks. As an example, 70.2% participants indicated their knowledge of depressive disorders was good or excellent and more than 70.0% of the participants indicated their work ‘sometimes’ or ‘often’ involved taking a mental history and doing a mental health examination in patients suspected of having a depressive disorder. However, the proportion of participants that felt quite or very confident in taking a mental health history or carrying out a mental health examination were only 50.3% and 43.2%, respectively. While 73.8% of the participants indicated their work sometimes or often involved providing counselling to patients with a depressive disorder, the proportion of participants who indicated they were quite or very confident performing this task was only 51.5%. While 59.8% of participant indicated that their current work sometimes or often involved prescribed pharmacological treatment to patients with a depressive disorder, only 46.1% of the participants indicated they were quite or very confident to pharmacological treatment to a patient with a depressive disorder. These confidence ‘deficits’ were also present to varying extents for anxiety disorders, substance use disorders, schizophrenia and suicide risk and suggest specific areas that need to be addressed in training.

In addition to strengthening training, changes in national policy may be required to use clinical associates more effectively in providing mental health services. The *Mental Health Care Act, 2002* predates the development of this cadre and, therefore, does not mention clinical associates in their definition of ‘mental health practitioner’ which currently includes psychiatrists, medical doctors, nurses, psychologists, occupational therapists, and social workers.^39^ An amendment to the Act to include clinical associates should be considered particularly as one of the two mental health practitioners who needs to examine mental health care users during the application for involuntary care needs to be qualified to perform a physical examination and one of the two mental health practitioners responsible for 72 h observation of involuntary patients must be a medical practitioner.^39^ The formalisation of clinical associates as mental health practitioners could, therefore, reduce the clinical and administrative workload of medical doctors with respect to mental health patients. A further change in policy to be considered relates to the prescription of Schedule 5 medication by clinical associates. The current limitation could be eased based on additional training for example, a prescribing course for psychiatric medication.

### Limitations

We were reliant on PACASA and university alumni databases to reach potential participants. These may have been incomplete and/or lacked up to date contact information. We did not have access to a WSU alumni database and as a result clinical associates from WSU were underrepresented in our study. It is not clear how many clinical associates in total were reached and it is, therefore, not possible to calculate a response rate. We did, however, offer incentives, send out reminders and extend the closing date of the survey to encourage as many clinical associates to participate as possible. It is possible that the clinical associates who participated were those interested in mental health so generalisation to all clinical associates in South Africa should be done with caution. As our study relied on self-assessment of knowledge, this may have been overestimated by participants and the possibility of the Dunning-Kruger effect cannot be ruled out.^40^

## Conclusion

Clinical associates are currently involved in mental health assessment and management. Their self-assessed knowledge of mental illness was generally good. Based on their confidence scores, there appeared to be some gaps with respect to practically performing aspects of mental health assessment and management. There is an urgent need to provide additional training to close the gaps that exist among those clinical associates already involved in mental health care as well as strengthen undergraduate training in mental health. Consideration needs to be given to amending the *Mental Health Care Act, 2002* to include clinical associates in the definition of ‘mental health practitioner’ and formally allowing clinical associates with appropriate training to prescribe psychiatric medication.
